# An update on respiratory syncytial virus

**DOI:** 10.1186/s12879-023-08730-x

**Published:** 2023-10-27

**Authors:** Antonio Piralla, Zhengrong Chen, Hassan Zaraket

**Affiliations:** 1 Microbiology and Virology Department, Fondazione IRCCS Policlinico San Matteo, via Taramelli 5, Pavia, 27100 Italy; 2 Department of Respiratory Disease, Children’s Hospital of Soochow University, NO. 92, Zhongnan Street, Suzhou, PR of China; 3 Department of Experimental Pathology, Immunology, and Microbiology, Faculty of Medicine, American University of Beirut, Beirut, Lebanon

## Abstract

Respiratory syncytial virus (RSV) is a leading cause of acute respiratory infections resulting in a significant burden worldwide, particularly in children and older adults. This collection calls for original research papers that advance our understanding of the epidemiology, evolution, diagnosis, clinical management, and prevention of RSV infections.

Respiratory syncytial virus (RSV) is a leading cause of acute respiratory tract infection, including lower respiratory tract infection (LRTI). Before the coronavirus disease 2019 (COVID-19) pandemic RSV represented the fourth cause of overall disability-adjusted life-years at all ages [1]. The burden of RSV infection is highest in children aged < 5 years (global incidence 17.0 (95% uncertainty intervals (UI) 10.6–26.2) per 1000 people), older adults aged > 70 years (global incidence 6.3 (95% UI 4.9–7.8) per 1000 people) and adults with underlying comorbidities [2]. As well as being well-established as a pediatric pathogen, RSV infections have been increasingly reported in adults. In particular, the number of RSV cases in older adults in high-income countries has been estimated as high as 10.9 million, resulting in 0.8 million hospitalizations and as many as 74,000 deaths [3].

RSV annual epidemiology is also impacted by the alternation of the subtypes A and B and, within them, by genetic variations of strains or lineages [4]. Indeed, several studies demonstrated that RSV variability is higher than previously thought and impacted the number of RSV hospitalizations and clinical severity in pre-pandemic seasons. Subsequently, RSV genetic evolution could have been impacted by the COVID-19 pandemic [5]. The drop in infections could have caused a genetic bottleneck resulting in the extinctions of pre-pandemic lineages and the emergence of a reduced number of RSV strains, and/or local variations [4]. RSV, subtypes A and B, classification has been recently revised by several groups to unify criteria and nomenclature following the availability of a larger number of full-genome sequences, together with those of the G gene that has been traditionally used for genotype designation [5].

Before the COVID-19 pandemic, the detection of RSV infections followed a predictable seasonal pattern each year. During the first year of the pandemic, respiratory viruses, including RSV, caused an unusually low number of infections and related hospitalizations in the first phase of the COVID-19 pandemic, due to the implementation of non-pharmaceutical interventions [6]. The subsequent reduction of pandemic restrictions has caused the reappearance of RSV in summer-early autumn 2021 [7]. To explain the atypical inter-seasonal resurgence of respiratory infections around the world, the Pediatric Infectious Disease Group proposed the concept of an “immunity debt” [8]. In the following epidemic season, autumn 2022, the RSV epidemiology was again impacted by SARS-CoV-2 circulation, but also by the return of the influenza virus. The three viruses co-circulated together abundantly causing a heavy burden on healthcare services [9]. This occurrence defined as “a tridemic” was unexpected, given the previously demonstrated interference between SARS-CoV-2 and influenza virus, and RSV and influenza virus [9]. Indeed, viral interference is a complex phenomenon driven by viral properties and by host immunity. At the population level, viral competition for the same host can shape the circulation of seasonal and pandemic respiratory viruses [9].

Currently, treatment of RSV infections relies on supportive care including supplemental oxygen, rehydration, and mechanical ventilation when critical. Antiviral treatment with aerosolized ribavirin is limited to severe infections in immunocompromised patients [10]. Until recently, only palivizumab, a multiple-dose monoclonal antibody (mAb) has been available for immunoprophylaxis against severe RSV-related lower respiratory tract illness (LRTI) in premature and other high-risk infants [10]. More recently, nirsevimab, a longer-lasting, single-dose mAb for the general infant population (preterm and term infants) targeting the RSV fusion glycoprotein (F) was approved. Nirsevimab provides protection for a whole season and has an efficacy of 74.5% against medically attended RSV-LRTI and 62% against hospitalizations due to severe RSV-LRTI [11].

On the vaccine front, after decades of troubled RSV vaccine development, four randomized clinical trials in older adults and pregnant women were recently published revealing a breakthrough in providing a high level of protection against RSV [12–15]. The successful development of these vaccines was mainly enabled by structure-based design and research demonstrating that the F protein in its perfusion state (PreF) elicits high levels of potent neutralizing antibodies [14]. In a randomized clinical trial of 24,966 adults aged 60 and above, an adjuvanted stabilized PreF recombinant protein-based vaccine was associated with a 94% efficacy against severe RSV-related LRTI and 72% efficacy against RSV acute respiratory infection [15]. In another trial with 34,284 adults > 60 years old Walsh et al. also showed that a recombinant PreF protein-based vaccine resulted in similar high efficacies of 67% and 86% against RSV-associated LRTI with at least two or three signs or symptoms, respectively [13]. Falsey et al. demonstrated in a trial with 5782 adults aged 65 and above that an adenovirus-based PreF vaccine candidate led to 70–80% efficacy depending on the disease definition [14]. Finally, a bivalent PreF protein-based maternal vaccine offered 82% protection against medically attended RSV-associated LRTI in infants within 90 days after birth and 69% at 6 months after birth for severe RSV in their infants [12]. These studies were the basis of the FDA’s approvals of two vaccines (Arexvy and Abrysvo^TM^) for adults aged 60 and older and for pregnant women (Abrysvo^TM^) to protect infants from birth up to 6 months of age [16].

The recent advances and progress toward the prevention of RSV have re-energized the field and highlighted the need for continued research to better understand the disease as well as the epidemiology and evolution of RSV. Studies assessing the impact of the newly approved immunotherapeutic and vaccines on RSV burden and genetic diversity are critically needed. This collection calls for original research papers that aim to improve our understanding of RSV epidemiology, evolution, diagnosis, clinical management and prevention.

## Data Availability

Not applicable.
